# Conceptual causal models of socioeconomic status, family structure, family functioning and their role in public health

**DOI:** 10.1186/s12889-021-10214-z

**Published:** 2021-01-22

**Authors:** Frederik Booysen, Ferdi Botha, Edwin Wouters

**Affiliations:** 1 School of Economics and Finance, University of the Witwatersrand, 1 Jan Smuts Avenue, Braamfontein, 2050 Johannesburg, South Africa; 2 Melbourne Institute: Applied Economic & Social Research, The University of Melbourne, Melbourne, Australia; 3 ARC Centre of Excellence for Families and Children Over the Life Course, Melbourne, Australia; 4 Faculty of Social Sciences, University of Antwerp, Antwerp, Belgium

**Keywords:** Social determinants of health, Family structure, Family functioning, Socioeconomic status

## Abstract

Social determinants of health frameworks are standard tools in public health. These frameworks for the most part omit a crucial factor: the family. Socioeconomic status moreover is a prominent social determinant of health. Insofar as family functioning is poorer in poor families and family structure and functioning are linked to health, it is critical to consider the pathways between these four constructs. In this correspondence, we reflect on how empirical studies of this conceptual nexus mirror two causal models. We conclude by reflecting on future directions for research in this field.

## Introduction

Social determinants of health (SDH) frameworks are standard tools in informing researchers, practitioners and policymakers of the underlying role of social factors in improving public health [1]. The World Health Organization’s (WHO) Commission on the Social Determinants of Health defined SDH as “the conditions in which people are born, grow, live, work and age” and “the fundamental drivers of these conditions” [2]. The impact of these SDH on health outcomes have been repeatedly demonstrated [3, 4]. As a response, the WHO developed a ‘Conceptual Framework for Action on the Social Determinants of Health’. This framework outlines the *structural determinants* of health inequities both at the macro (socio-economic and political context) and micro (social class, gender and ethnicity) level. The model further stipulates that these SDH operate through a set of *intermediary determinants* of health, namely: (1) material circumstances; (2) psychosocial circumstances; (3) behavioural and/or biological factors; and (4) the health system itself [5]. However, despite the wealth of research on how health plays out in family and household contexts, we argue that theoretical discussions surrounding this framework for the most part omits explicit reference to a crucial but complex social factor that affects and is affected by health: the family.

## The family context

Individuals seldomly live in total isolation: the family context in which people eat, sleep and live could be both a structural determinant (family structure) as well as an intermediary determinant (family functioning) of health and even be affected by health. Families can have very different structures (nuclear, skip-generation, single parent, etc.), which have been shown to be a structural driver of the mental health of family members and especially children [6]. However, not only the structure of families can impact health. For families to fulfil their roles in society, families need to function well. Formally, family functioning encompasses the ability of the family to accomplish the tasks necessary to achieve its well-being, the ability to adapt to changing circumstances, and the ability to balance individual family members’ needs with those of the family system [7]. Theoretically, the relationship of family functioning with health is reciprocal and complex in nature. Ill health may adversely affect family functioning [8]. Health stressors may however also unlock the family’s resilience and result in improvements in family functioning [9]. Members of well-functioning families may also enjoy better physical and mental health due to the greater instrumental and emotional support available to them [10], whereas dysfunction may result in the maintenance of poor health behaviours [11].

The body of empirical evidence on health and family functioning documented in reviews provide considerable support for claims of such an association, including in regard to overweight and obesity in children and adolescents [12], disability in children [13] and pediatric organ transplants [14]. A recent meta-analysis has found evidence of a significant association between less family conflict and greater family cohesion, expressiveness and support, and adjustment in pediatric cancer patients and their siblings [15]. Other recent meta-analyses suggest that the psychological health of children with chronic conditions is associated with many dimensions of family functioning, in particular conflict and cohesion [16], as is the case for medical adherence, especially regarding better problem-solving, positive communication, greater cohesion and flexibility, and less conflict [17]. Yet, in other instances, evidence has been lacking or mixed, including in regard to eating disorders [11], service use by young people with mental health issues [18], and childhood cancer [9]. The most prominent multi-dimensional measures of family functioning employed across these studies, which are grounded in diverse theoretical frameworks, include the McMaster Family Assessment Device (FAD), the Family Assessment Measure (FAM), the Family Environment Scale (FES) and the Family Adaptability and Cohesion Evaluation Scale (FACES) [11–14, 16].

Only recently, however, have scholars proceeded to explicitly conceptualise the family’s role as a SDH [19, 20]. One proposed approach has been to consider family structure as a structurally-determined and socially stratified grouping [21]. Another possibility, in respect of family functioning, is to consider it among the intermediary determinants as a specific psychosocial factor. There is evidence, moreover, albeit mixed, that poorer family functioning is associated with family structure, including family intactness and family configurations such as single parenthood and cohabitation [22–33].

## Proposed conceptual causal models

Socioeconomic status (SES), moreover, conceptualised broadly, is a prominent SDH. Insofar as family functioning has been shown to be poorer in families with lower SES [34–38], it is critical to consider the pathways between these constructs, i.e. socioeconomic status, the family (composed of family structure and functioning) and health. Drawing on the work of Wu and Zumbo [39], we offer some suggestions in the form of two sets of simplified causal models.

**Fig. 1:**
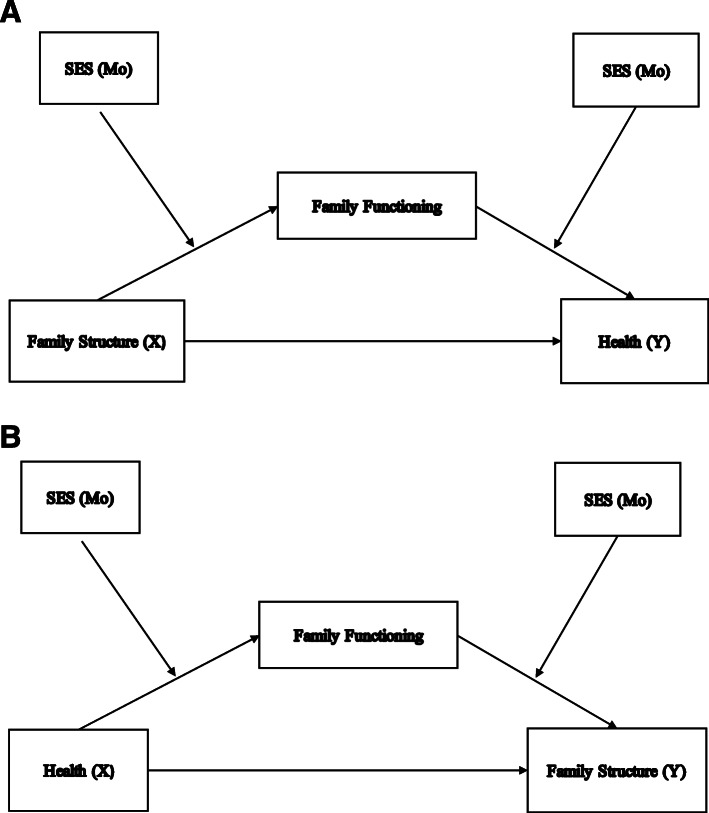
**a**: A moderated mediation model with health as an outcome. Note: Adapted from Fig. 6 [39]. **b**: A moderated mediation model with health as a determinant. Note: Adapted from Fig. 6 [39]

**Fig. 2:**
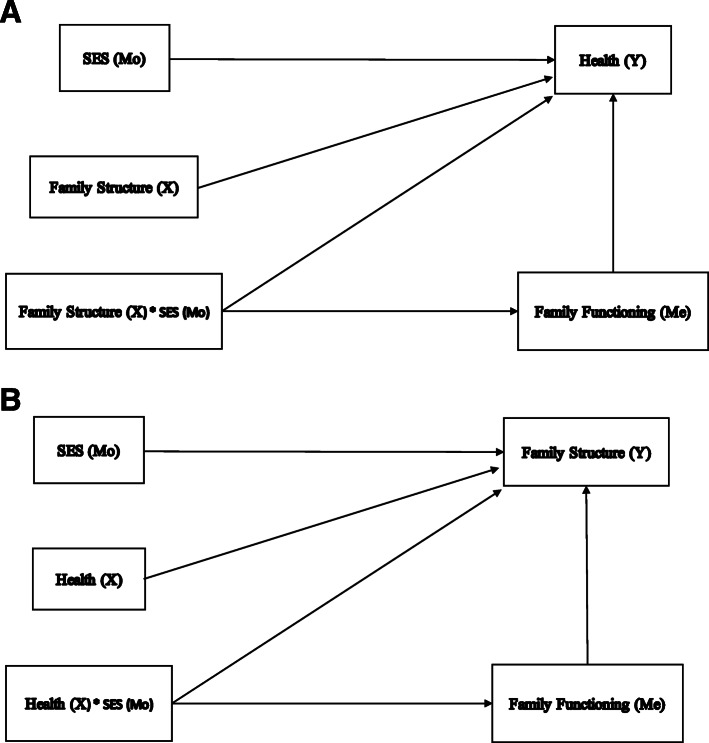
**a**: A mediated moderation model with health as an outcome. Note: Adapted from Fig. 7 [39]. **b**: A mediated moderation model with health as a determinant. Note: Adapted from Fig. 7 [39]

Assuming that the focus, on the one hand, is on the causal link between family structure (X) and health (Y), we could describe family functioning as a potential mediator (Me) that answers the question as to how and why family structure impacts on health. In turn, SES (Mo) may moderate both the causal link between family structure and family functioning as well as the causal link between family functioning and health (Fig. 1a). Family functioning may also mediate the causal interactive effect between family structure and SES (Fig. 2a).

On the other hand, Fig. 1b and Fig. 2b for each causal model recognises that health may also impact family functioning and family structure and that socioeconomic status may moderate these effects. Caution is required however insofar as family structure in the longer term is fluid too and that family functioning may be a function of family structure. The implication is that family structure may also be treated as a mediator rather than a moderator. The same applies to SES, which underlines the fact that further efforts at building appropriate and comprehensive causal models are required to advance this research agenda.

## Empirical work

Using a selection of the few studies that have focused on the family structure-family functioning-health nexus, we reflect on how this research mirrors these two exemplars of a causal model. In a recent study, Donley et al. [40] apply regression analysis to cross-sectional data collected from the parents of American children with special needs. The authors conclude that socio-economic factors are less impactful on delayed care than family dynamics. Yet, the SES and family functioning variables are included in two separate regression models rather than jointly entered into the same model, with no moderator analysis for SES and without taking family structure into account. In another of these studies, Sawyer et al. [41] applied regression analysis to cross-sectional data collected from a sample of parents of Australian children with asthma to determine the association of family structure (single versus two-parent families) and family functioning with health-related quality of life. Both family structure and functioning, treated as independent covariates, were found to be associated with mental health, while only family structure was associated with physical health and social functioning. Cheng et al. [42], in another recent study, examined the effects of family structure and functioning on mental health using regression analysis of cross-sectional household survey data from China. The authors found that only family functioning was associated with mental health and not family structure. Pless et al. [43], in a much earlier cross-sectional study on psychological adjustment in American school children with chronic physical disorders, also treated family structure and family functioning as independent covariates and SES as a control. Although individually significant, these authors did not explore the potential interaction between family structure and functioning. Wagner et al. [32], however, employ a longitudinal design in which the study outcome (substance use among American adolescents) was observed a year after family structure, functioning and other covariates, including SES. Using a Structural Equation Model (SEM), the authors do explore the nature of the mediating relationship in Fig. 1a and Fig. 2b and found that parental monitoring mediated the association of parental family structure with substance use. Again, however, as was the case in each of the other studies, SES was simply treated as a controlled covariate rather than a potentially important moderator of the family’s role as a SDH.

Among the handful of studies that we could locate that explore the impact of child and adult health on family functioning [8, 44–47] and family structure [48], which found that health negatively impacted family functioning [44–47] and modified family structure [48], SES generally is entirely omitted from the multivariate analysis [8, 44, 47, 48]. The only exception is Treyvaud et al. [46], who incorporated a measure of SES (employment status) into a multi-dimensional index of ‘social risk’, which was then employed as a controlled covariate.

## Future directions

To inform the development and evaluation of interventions, it is necessary to elucidate the wider causal mechanisms through which specific dimensions of family functioning are impacted by and acts on health and health-related behaviours, and how these dynamic processes play out over the life course and across generations in families with different structures. A first step in this endeavour is to build more nuanced and integrated causal models of the complex mechanics of the interplay between the constructs of socioeconomic status, family functioning, family structure and health, using the conceptual causal models presented here as a starting point. The second step is to develop and implement longitudinal research designs that will yield the necessary empirical data to test these theories, either comprehensively or more realistically in a piecemeal fashion, in both clinical but also general populations, scaling this research to more representative levels to allow a public health perspective. A key component of this part of the research agenda includes the development of multi-dimensional short-form scales of family functioning with good psychometric properties for use in general populations. Also, researchers need to consider collecting information on the perceptions of family functioning from all family members in the defined family structure [49]. Such assessments are often disparate [49] and measures of such discrepancy may themselves warrant consideration as a measure of family functioning. In each case, such theoretical and empirical research endeavours should be appropriately contextualised, including cross-culturally. The final step would be to apply state-of-the-art techniques for longitudinal analysis to these data. Given that all four these central variables co-evolve over time, latent growth modelling is of particular importance [50], as are autoregressive latent trajectories models [51]. There is also scope for expanded analyses of the valuable data collected in the various studies already conducted in this field to help further elucidate the complexities of these relationships. Such endeavour is inter- and multi-disciplinary at its core, requiring social scientists such as economists and sociologists to join health scientists and statisticians in demystifying the family’s role as a key SDH and health consequence.

## Data Availability

Not applicable.

## Abbreviations

SDH: Social determinants of health
SEM: Structural equation model
SES: Socioeconomic status
WHO: World health Organization
